# Hints and Improvements in the Practice of Medicine and Surgery

**Published:** 1799-12

**Authors:** 


### HINTS AND IMPROVEMENTS

### IN THE PRACTICE OF

### MEDICINE AND SURGERY.

### *On the Origin and Prevention of ſome of the Diſeaſes of Human Teeth and Bones.* By Dr. MITCHILL, *of New-York.*

[Concluded from p. 380—382 of our l.ſt Number.]

THE fœtor of the breath has an analogy, in ſuch caſes, with the offenſiveneſs of matter diſcharged from deceaſed bone, which, in general, is diſcoloured, and not thick; owing to a ſeparation of the phoſphoric acid of the bone from its calcareous baſe, now combined with ſeptic acid, and running away with the other fluids, in the form of a thin and ſanious diſcharge; or occaſionally bringing on, when abſorbed, that form of quotidian intermitting fever, called the *hectic*, as in other caſes of abſceſs and ulcer.

RUSSEL conſiders necroſis, whether happening in the tibia, femur, lower jaw, clavicle, humerus, fibula, radius, or ulna, as the ſame kind of diſeaſe, (p. 86.) If then, his claſſification of morbid affections be correct, and my application of principle to explain the phenomena be accurate, it will be proved, that the ſame ſeptic poiſon which deſtroys the teeth, corrupts the jaw; and from the ſame cauſe which diſorganizes the jaw, proceeds the decay of the reſt of the bones; and this ſame agent, which works the deſtruction of the reſt of the bones, is the irritating matter that kindles up hectic fever.

I would not wiſh to be underſtood as affirming that *all* caries of the teeth, jaws, and bones, ariſe from this ſole cauſe. Far from it. The amount of my reaſoning is ſimply this; that, from the moſt accurate ſurvey I have been able to take of the ſubject, there does appear to be, in *ſome inſtances*, a decompoſition of bone, by means of ſeptic acid, abſorbed from without, [or formed, by union of ſepton with oxygen, within the conſtitution; and when this acid, mingled with other animal fluids, is carried into the blood-veſſels, and exerts its noxious powers ers upon the heart, brain, and lungs, it may be the cauſe of febrile inquietude.

How far this principle may extend, if properly applied, I know not. I ſuſpect that *ſyphilitic*, *cancerous*, and *ſcrophulous* ulcerations will be found to have a near alliance, as to their cauſe, with caries of teeth and necroſis of bone now under conſideration. But time, with farther obſervation and experiment, is neceſſary to refute or verify this conjecture. Be the reſult as it may, I think, as the game is ſtarted, and the track is freſh and warm, it would betray leſs than a ſportſman's ſpirit to be diſcouraged, on account of the doublings and windings of the chace, and give out before the object of purſuit is hunted down, or day-light let in upon its dark abode.

---

### *On the Dolor Faciei, obſerved by Fothergill.*

It is generally believed that this diſeaſe has been ſcarcely, if it all, known to practitioners, before the late Dr. Fothergill deſcribed its nature and medical treatment.

In the Tenth Number of the "*Journal der Erfindungen*," however, we meet with the following account relative to this ſubject: Prof. Siebold, jun. of Würzburg, ſhewed in a Treatiſe, entitled, "*Doloris Faciei, morbi rarioris, atque atrocis obſervationibus illuſtrati adumbratio*, 4to. 1795; that this ſingular diſeaſe was obſerved and deſcribed by Bausch, the founder and firſt preſident of the *Acad. Natur. Curioſor.* who died in the year 1655. This writer was himſelf ſubject to the diſeaſe, and gave an account of it, in the *Ephemer. Nat. Cur. Dec.* I. Ann. II. where he delivers the hiſtory of the academy. A ſimilar caſe occurs in the next volume of theſe Ephemerides, p. 455; but the moſt accurate and complete deſcription of this painful affection is furniſhed by Degner, in the *Act. Natur. Cur.* Vol. I. p. 347—half a century prior to Fothergill. A French ſurgeon of the name of Andre likewiſe obſerved the diſeaſe in the year 1756, and gave it the name of *tic*.

"After having mentioned all the modern writers on the ſubject, Prof. Siebold adds, that he himſelf has obſerved the *dolor faciei* in a female, without being able to trace any cauſe of that complaint. The patient was ſucceſſively treated with cicuta, bark, and valerian, while veſicatiories and mercurial frictions were applied externally: ſhe at length tolerably recovered."

 The

The reviewer of this article, in the Journal above mentioned, informs us, that he has only once met with the troublesome malady, which the Germans call "*the Fothergillian face-ach.*" His patient was a lady of a sound constitution, about forty years of age, and mother of several children. She had been tormented with this pain for eighteen months, during which time she submitted to the ineffectual treatment of several physicians, as no determinate cause of it could be discovered. He was at length induced to prescribe as follows:— ℞. Pulv. Folior. Belladonn. gr. v. Rad. Rhei gr. iij, Sacchar alb. ℨss. M. D. tales dos. No. viii. One of these powders was directed to be taken every other night; and this obstinate case, according to the German account, yielded completely to that remedy!

